# Framework for Groove Rating in Exercise-Enhancing Music Based on a CNN–TCN Architecture with Integrated Entropy Regularization and Pooling

**DOI:** 10.3390/e27030317

**Published:** 2025-03-18

**Authors:** Jiangang Chen, Junbo Han, Pei Su, Gaoquan Zhou

**Affiliations:** 1 College of Sports and Health Sciences, Xi’an Physical Education University, Xi’an 710068, China; 2 School of P. E and Sports, Beijing Normal University, Beijing 100875, China

**Keywords:** music perception, groove rating, convolutional neural networks, temporal convolutional networks, entropy regularization, entropy pooling

## Abstract

Groove, a complex aspect of music perception, plays a crucial role in eliciting emotional and physical responses from listeners. However, accurately quantifying and predicting groove remains challenging due to its intricate acoustic features. To address this, we propose a novel framework for groove rating that integrates Convolutional Neural Networks (CNNs) with Temporal Convolutional Networks (TCNs), enhanced by entropy regularization and entropy-pooling techniques. Our approach processes audio files into Mel-spectrograms, which are analyzed by a CNN for feature extraction and by a TCN to capture long-range temporal dependencies, enabling precise groove-level prediction. Experimental results show that our CNN–TCN framework significantly outperforms benchmark methods in predictive accuracy. The integration of entropy pooling and regularization is critical, with their omission leading to notable reductions in R^2^ values. Our method also surpasses the performance of CNN and other machine-learning models, including long short-term memory (LSTM) networks and support vector machine (SVM) variants. This study establishes a strong foundation for the automated assessment of musical groove, with potential applications in music education, therapy, and composition. Future research will focus on expanding the dataset, enhancing model generalization, and exploring additional machine-learning techniques to further elucidate the factors influencing groove perception.

## 1. Introduction

Groove represents a complex and multifaceted aspect of music perception. The perceptual groove in music is characterized by its capacity to evoke both emotional and physical responses in listeners [1] and is integral to the essence of groove [2]. Madison was among the pioneers in defining groove [3], and subsequent work by Janata et al. [1] further expanded the concept into a structured psychological framework. Currently, a widely accepted definition of groove encompasses the ability to perceive the beat, recognize recurring rhythmic patterns, and elicit synchronized motor responses, such as foot tapping and dancing in time with the beat [4], as well as to derive pleasure from these activities [5,6]. Given its role in driving physical movement and synchronization, groove is widely incorporated into exercise music to enhance motor performance [1,2].

Groove holds considerable importance in domains such as music education, therapy, and composition. Research into the impact of groove on education, motor skills, and rehabilitation presents significant potential [7]. However, the precise quantification and prediction of groove remain challenging. In previous studies, Janata et al. made a substantial contribution by employing behavioral and computational methods to quantitatively assess the degree of sensorimotor coupling, thereby evaluating the perceptual depth of groove. They curated a playlist accompanied by comprehensive groove ratings, which has since been used as a valuable tool in subsequent research. These studies have validated its applicability in real-world scenarios [8,9,10]. However, the process of determining groove through behavioral metrics is complex, and this framework is difficult to scale. As a result, the original playlist, containing a limited selection of music, has been in use for over a decade without significant updates. Consequently, later studies have often had to rely on outdated musical selections, highlighting the urgent need for a method that allows for the rapid and large-scale assessment of groove. This necessitates an approach that decodes the intrinsic musical attributes of groove, thereby enabling its rating and classification.

The challenge now lies in the quantification and large-scale prediction of groove, which requires new methodologies beyond traditional behavioral experiments. Since previous studies have established that groove is closely related to intrinsic musical attributes, such as syncopation, rhythmic complexity, and spectral features like timbre and harmony, there is potential to leverage these attributes for groove prediction [11,12]. Syncopation, for example, plays a crucial role in triggering anticipatory rhythmic sensations, while rhythmic complexity balances predictability and deviation in music [13]. However, the large number of acoustic features and the complex nonlinear relationships between them make traditional regression methods insufficient for effective modeling and predicting groove. Fortunately, machine-learning techniques offer the capacity to handle such high-dimensional and nonlinear features, providing a promising and feasible approach to investigate the potential of these acoustic attributes in groove prediction [14]. By employing machine learning, we can uncover latent relationships among complex features, leading to more accurate prediction and quantification of groove.

In light of the limited size and scope of the existing dataset, this paper pivots its focus to the evaluation of the effectiveness of entropy regularization and entropy-pooling techniques within the current dataset. By integrating these techniques into the Convolutional Neural Network (CNN) and Temporal Convolutional Network (TCN) architecture, we aim to enhance the learning process and model performance.

During the preprocessing stage, audio files are converted into Mel-spectrograms and then fed into the CNN for feature extraction. The TCN, with its causal convolution layers, is employed to capture long-range dependencies in the music’s temporal sequences. Notably, within the CNN component, the traditional global average pooling layer has been replaced with an entropy pooling layer. This substitution not only reduces the model’s sensitivity to extreme values but also cuts down on computational resource consumption. Additionally, entropy is introduced as a regularization term in the loss function, further optimizing the model.

The main contributions of this study are as follows:

1. We conduct an in-depth evaluation of how entropy regularization and entropy-pooling techniques improve the learning process within the existing dataset when integrated into the CNN–TCN architecture for music groove rating.
2. We present empirical evidence highlighting the significant role of these entropy-based techniques in enhancing the model’s performance, including its generalization ability and stability, despite the limitations of the dataset.

This research approach allows us to explore the potential of entropy-related methods in improving model performance without the need for a large-scale labeled dataset, providing valuable insights into music groove rating within the constraints of the available data.

## 2. Related Works

In this section, we review methodologies for rating groove perception in music, with a particular focus on both conventional and deep-learning approaches. Given that research specifically focused on predicting groove may be relatively limited, we also review studies on emotional response prediction, as these studies often use acoustic features as predictive variables, offering methodological insights that can be adapted for groove prediction.

### 2.1. Methodologies for Predicting Perceived Groove in Music

Hove et al. [15] identified acoustic features that influence groove through behavioral experiments. Their findings revealed that enhancing low-frequency components of music, as well as perceiving these frequencies through tactile means, significantly promotes groove, thereby establishing the impact of specific acoustic characteristics on groove perception. Madison et al. [16] further investigated the influence of other acoustic features on groove, utilizing linear regression analysis. Their study highlighted beat salience as a significant predictor of groove. In another study, Stupacher et al. [4] examined a broader range of acoustic features in relation to groove, employing correlation analysis. They found that variability in event density is also a strong predictor of groove.

However, these studies face several unresolved challenges. First, there is potential for improving decoding accuracy. In Stupacher’s research, the highest correlation between acoustic features and groove level was 0.51 [4], while in Madison’s study, the highest correlation reached 0.61 [16]. Additionally, neither Stupacher nor Madison utilized nonlinear approaches, such as machine learning or deep-learning methods, which limits their models’ ability to capture complex relationships and provide comprehensive summaries of nonlinear interactions.

### 2.2. Methodologies for Recognizing Emotional Responses to Music

The methodologies for analyzing groove perception share important technical commonalities with emotion recognition research. Both domains have employed regression and machine-learning techniques, though groove analysis specifically focuses on rhythmic entrainment and embodied responses. Nonlinear approaches like neural networks demonstrate particular promise for both domains by capturing complex temporal patterns. Notably, several emotion recognition techniques we examined inspired our methodological choices for groove prediction.

Mori et al. [17] demonstrated the limitations of linear regression (43% accuracy) for music-induced physiological-response classification. This finding motivated our exploration of nonlinear alternatives for groove prediction. Daly et al. [18] showed feature combination benefits (correlation 0.234) using linear regression, reinforcing our decision to employ multi-modal feature integration.

Of particular relevance to our technical approach, Vempala et al. [19] demonstrated neural networks’ superiority over linear methods (lower RMSE) for valence/arousal prediction. Their three-layer architecture informed our baseline model design, though we extended it with entropy regularization techniques. Weninger et al. [20] achieved R² = 0.50–0.70 using LSTM networks, prompting our initial experimentation with recurrent architectures for groove modeling. However, we found LSTMs less effective for rhythmic pattern capture compared to CNN-based approaches.

Keunwoo et al. [21] then used transfer learning techniques. They used OpenL3, a pre-trained deep-learning model [22], to extract high-dimensional 6144-dimensional feature vectors from music data. These features are less interpretable than traditional acoustic features. Then, a variant of Support Vector Machines (SVM) was used for classification and regression related to valence and arousal. The performance was comparable to Weninger et al. [20], with improvements in computational efficiency and robustness. The use of OpenL3, a CNN, showed that CNNs can capture global and local features from music spectrograms, which have more potential than traditional acoustic feature-extraction methods. However, the SVM variant had trouble with the high-dimensional, time-series nature of the data, limiting the model’s overall performance. While we adopted their CNN feature-extraction paradigm, we replaced the SVM with deep networks better suited for temporal modeling. Crucially, we introduced entropy regularization to address the high-dimensional feature challenge they identified.

### 2.3. Entropy-Aware Learning Techniques

Entropy regularization originates from information-theoretic principles, first formalized by Grandvalet and Bengio in semi-supervised learning [23]. These principles were later extended through the information bottleneck framework [24], optimizing the balance between feature compression and prediction fidelity. Recent developments demonstrate entropy’s versatility in audio processing, as shown by the maximum entropy model achieving robust audio classification under complex acoustic conditions [25].

In music perception research, entropy-based approaches address critical modeling challenges. Wang et al. [26] developed an entropy-guided fusion method for multi-modal emotion recognition, integrating acoustic and visual features through entropy-weighted predictions. This approach achieved state-of-the-art performance in the MER-2023 challenge. Similarly, Ronnie et al. [27] pioneered music genre classification using Shannon entropy and Kullback–Leibler divergence of amplitude variations, attaining 82% accuracy across multilingual music datasets. Their frame-level entropy analysis revealed consistent patterns: melodic songs exhibit 23% lower entropy values than rock genres, enabling language-agnostic classification.

These methodologies directly inform our groove prediction framework, such as entropy pooling and entropy regularization, which could potentially accelerate model convergence and improve robustness.

## 3. Methodology

### 3.1. Overall

**Figure 1:**
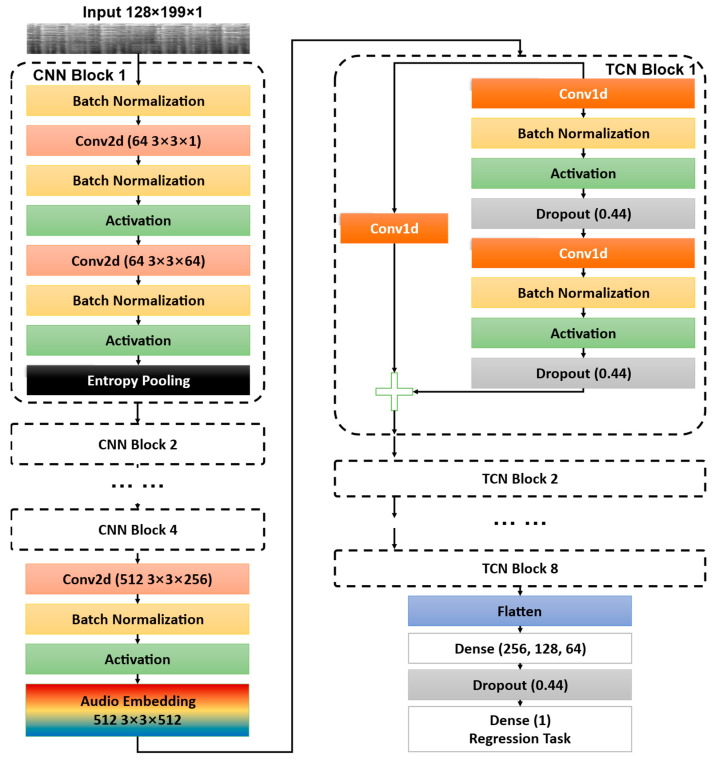
Overall structure of the model.

The overall structure of the CNN–TCN model is illustrated in Figure 1. The process begins with the input of a Mel-spectrogram, which represents the audio signal in the Mel frequency scale, providing a perceptually relevant representation of sound [28]. The Mel-spectrogram is particularly effective for mimicking human perception of different frequencies [29]. In the CNN processing stage, the input Mel-spectrogram is first normalized and then passed through a series of 2D convolutional layers. Each convolutional layer is followed by a ReLU activation function and an entropy-pooling layer. This sequence of convolution, activation, and entropy pooling is repeated multiple times, with each layer progressively refining the feature maps. These features are then embedded through a specialized convolutional layer, producing a three-dimensional array representing the samples, time steps, and feature maps.

These extracted features are subsequently passed to the Temporal Convolutional Network (TCN) for further processing. The TCN consists of multiple blocks, each with a similar structure, including a 1D convolutional layer, layer normalization, ReLU activation, dropout, and residual connections. These blocks are designed to capture temporal dependencies in the data. Finally, the outputs of the TCN blocks are fed into a fully connected layer, which produces the final output for regression tasks.

### 3.2. Two-Dimensional Convolutional Layers

Convolutional Neural Networks are powerful tools for extracting spatial features from grid-like data, such as images or spectrograms [30]. In this model, CNNs are used to analyze the Mel-spectrograms of audio signals, capturing both time-frequency patterns that are essential for understanding the characteristics of music.

The CNN layers in our model are designed to progressively refine the input Mel-spectrogram through a series of convolutional operations. Each 2D convolutional layer applies a set of filters to the input data, generating feature maps that highlight specific patterns in the spectrogram. The operation of a 2D convolutional layer can be mathematically described as follows:

$$F_{l+1}=\sigma (W_{l}*F_{l}+b_{l})$$

where:

- $F_{l+1}$ represents the input feature maps at layer *l*,
- $W_{l}$ is the weight matrix (filters) for the convolutional layer,
- ∗ denotes the 2D convolution operation,
- $b_{l}$ is the bias term,
- σ(⋅) represents the activation function, typically a ReLU in this context.

The CNN structure is built to extract increasingly abstract features from the Mel-spectrogram as the data passes through successive layers. Early layers might capture basic patterns such as edges or harmonics, while deeper layers identify more complex features like rhythmic structures or timbral characteristics.

### 3.3. Temporal Convolutional Network

The Temporal Convolutional Network extends the capabilities of CNNs to handle sequence data, making it particularly suitable for tasks involving temporal patterns, such as music analysis [31]. TCNs have demonstrated superior performance over traditional recurrent networks, such as Long Short-Term Memory (LSTM) networks, across a wide range of sequence modeling tasks due to their ability to capture long-range dependencies and their computational efficiency [32].

A key feature of TCNs is the use of causal 1D convolutions, which ensure that the model’s predictions at any time step depend only on current and past information, preserving the temporal order. This is crucial for tasks like music groove rating, where future information should not influence the interpretation of the current time step.

TCNs use dilated convolutions to expand the receptive field without increasing the computational load. The dilation factor grows exponentially with the depth of the network, allowing the model to cover a broader temporal context efficiently. The operation of a 1D dilated convolution for an input sequence $x\in R^{T}$ and a filter *h* of length *k* is defined as:

$$H(s)=(x*d_{d}h)(s)=\sum_{i=0}^{k-1}h(i)\cdot x_{s-d\times i}$$

where:

- ∗d represents the convolution operation with a dilation factor *d*,
- s is the position in the sequence,
- $d=2^{v}$, where *v* is the network depth level,
- k is the filter size, and
- s−d×i represents the temporal offset, ensuring that only past information is considered.

By increasing the dilation factor d with network depth, the receptive field grows exponentially, allowing the model to incorporate long-range temporal dependencies. This is particularly beneficial in music groove rating, where understanding rhythmic structures over extended time periods is crucial.

Each TCN block in our model comprises multiple dilated convolutional layers, followed by a rectified linear unit (ReLU) activation function, spatial dropout, and layer normalization to prevent overfitting and enhance generalization. Residual connections are incorporated to facilitate gradient flow during backpropagation, ensuring stable training of deep networks. The output of each block is defined as:

O=activation ⁡(x+F(x))

where:

- x is the input to the block,
- F(x) represents the sequence of transformations (dilated convolutions, ReLU, dropout) applied to x,
- The activation function is typically ReLU.

The residual connections in each block effectively double the receptive field size, enabling the network to capture intricate temporal dynamics in the music data.

This combination of dilated convolutions, causal convolutions, and residual connections allows the TCN to model complex temporal patterns in music, making it an ideal choice for tasks such as groove rating, where capturing the nuanced timing relationships in the audio is essential.

### 3.4. Entropy-Pooling Layer

The entropy pooling layer is a critical component that enhances the model’s ability to focus on informative features. This layer operates by computing the entropy of the feature maps, which measures the uncertainty or information content in each feature [33]. The pooling operation is guided by this entropy, emphasizing features with higher information content. The entropy of a feature map can be calculated as:

$$H(F)=-\sum_{i=1}^{N}p_{i}log(p_{i})$$

where:

- $p_{i}$ represents the probability distribution over the feature map values, typically derived using a softmax function,
- N is the total number of elements in the feature map.

The pooled output is then computed by weighting the original feature map values by their corresponding entropy values:

$$P=\sum_{i=1}^{N}H(F_{i})\cdot F_{i}$$

where P is the pooled feature map, which is passed to the subsequent layers. This mechanism ensures that features with higher uncertainty (and potentially higher importance) are retained more prominently in the model’s subsequent layers.

### 3.5. Entropy-Enhanced Loss Function

The final loss function used in this model combines the standard mean squared error (MSE) loss with an entropy-based regularization term. This helps the model not only minimize the prediction error but also manage the uncertainty in its predictions [34]. The loss function can be expressed as:

L=1N∑i=1N(y^i−yi)2+λ⋅1N∑i=1NH(Fi)

where:

- y^i is the predicted value,
- $y_{i}$ is the true value,
- N is the number of samples,
- λ is a regularization parameter controlling the weight of the entropy term,
- $H(F_{i})$ is the entropy of the output feature map.

The entropy term acts as a regularizer, encouraging the network to maintain a balance between certainty and flexibility in its predictions. This combination allows the model to achieve robust performance in rating the groove in music.

Through the above process, we can effectively rate the groove in music tracks and optimize the network parameters through backpropagation to achieve accurate and reliable groove ratings.

## 4. Experiments

### 4.1. Settings

#### 4.1.1. Datasets

The music clips used in this study were sourced from songs listed in the appendix of Janata’s 2012 research [1]. These tracks span various genres, including jazz, folk, rock, soul, funk, and R&B. MIDI drum loops from the original study were excluded, as they do not constitute complete songs. Music resources were acquired via iTunes, culminating in a final dataset of 86 songs. Some tracks were excluded due to the unavailability of corresponding resources, primarily due to their age, while others were omitted for lacking complete drum arrangements, following the precedent set by previous studies. The selected 86 songs were processed using the Python library “pydub”(Version 0.25.1). This processing involved trimming the first and last 30 s of each track and randomly selecting three non-overlapping 25-s clips from the remaining content, resulting in a total of 258 music clips for subsequent analysis.

Groove ratings were also sourced from the appendix of Janata et al.’s 2012 study [1]. In that study, 19 undergraduate students quantified groove using a 128-point scale, where higher scores corresponded to higher groove ratings. The 128-point scale was chosen due to the use of MIDI sliders, which have 128 pitch levels, for quantification.

#### 4.1.2. Baselines

In the current body of research, the highest accuracy in predicting musical characteristics has been achieved in predicting valence and arousal, with the model attaining an R^2^ of 0.500 for valence prediction and an R^2^ of 0.704 for arousal prediction [20]. We conducted a *t*-test using SPSS 24 to compare the difference between the mean R^2^ and the baseline R^2^ value, which was set at 0.704, based on the highest reported R^2^ in previous studies.

#### 4.1.3. Implementation Details

The network was implemented using the TensorFlow library in Python (Version 2.10.0, Python 3.9.17). The number of blocks, the receptive field of convolutional kernels, the dropout rate, and the number of neurons in the fully connected layer were optimized using the Optuna library in Python [35]. We conducted 1000 optimization trials, with each trial involving 200 epochs of training. Model performance was evaluated based on the mean squared error (MSE) on the test dataset, and the best set of hyperparameters from the 1000 trials was selected as the final configuration. The model was trained using the SGD+M optimizer with a batch size of 32. Training was conducted over 1000 epochs, with performance continually assessed based on the MSE on the test dataset. A checkpoint callback was employed to save the model weights when the MSE on the test dataset reached its minimum value. To assess the model’s performance, we employed a 5-fold cross-validation approach. The dataset was divided into five equally sized subsets. The model underwent five rounds of training and evaluation. Model performance in each round was assessed using metrics such as MSE and R^2^. Finally, we computed the average performance metrics across the five validation rounds.

### 4.2. Experimental Results

**Figure 2:**
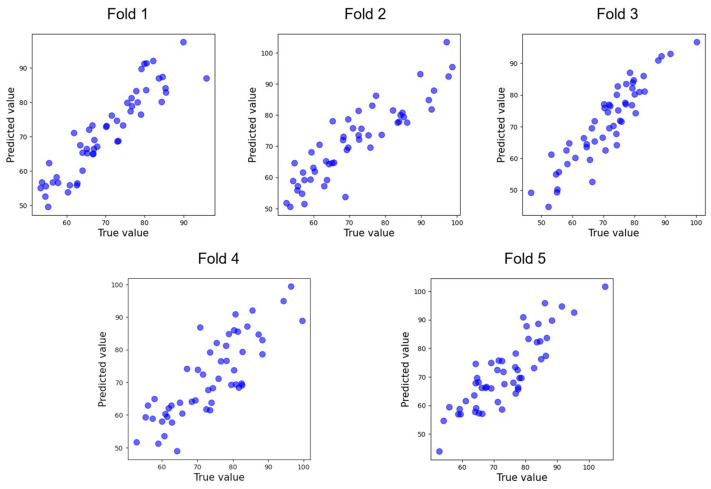
Scatterplot of per-fold validation.

As shown in Table 1, after 5-fold cross-validation, the model achieved an average MSE of 85.177 and R^2^ of 0.817. Scatter plots for each fold are displayed in Figure 2. The result of the one-sample t-test indicates that the R^2^ value of the model is significantly greater than the baseline value of 0.704 (t = 9.515, *p* = 0.000).

### 4.3. Comparison with Other Methods

In Table 2, we conducted a comparative analysis of different methods for predicting groove levels. First, we examined the impact of entropy pooling and regularization on prediction accuracy. When only entropy pooling was applied, the R^2^ decreased by 1.3% compared to the proposed method. Similarly, using only entropy regularization resulted in a 3.4% decrease in R^2^. When neither entropy pooling nor regularization was applied—replacing them with average pooling and a conventional loss function—the R^2^ decreased by 5.1%.

**Figure 3:**
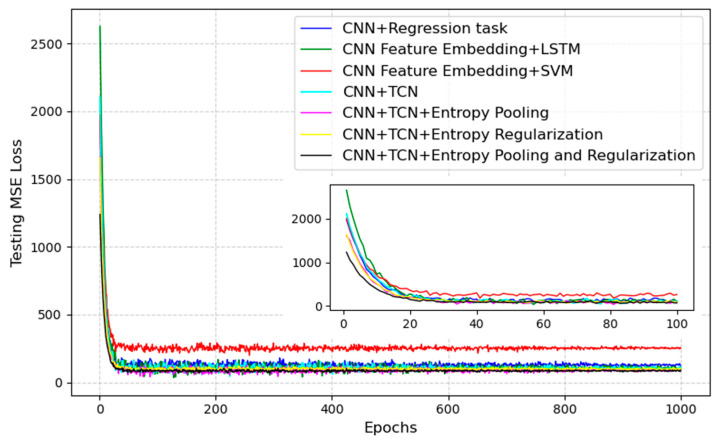
MSE of the testing set for each algorithm during the training process.

Additionally, we compared the accuracy of directly performing regression tasks following the CNN. This approach resulted in an 8.6% reduction in R^2^ compared to the method proposed in this study. Furthermore, when the features extracted by the CNN were used for regression tasks via LSTM or a variant of SVM, the R^2^ decreased by 11.0% and 26.9%, respectively, compared to our proposed method. Figure 3 shows the MSE of the test set for each algorithm during the training process.

**Figure 4:**
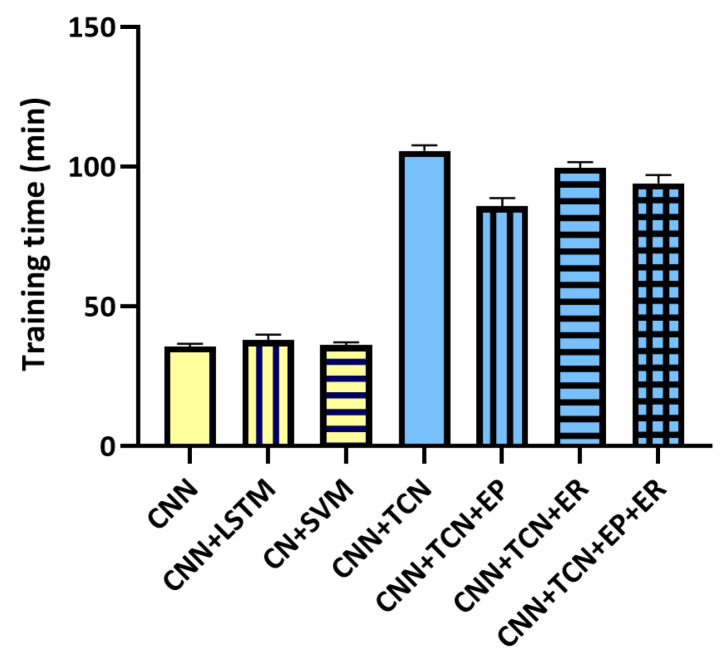
Comparison of training time for different models; Note: Abbreviation: CNN: Convolutional Neural Network; TCN: Time Convolutional Neural Network; LSTM: Long short-term memory; SVM: Support Vector Machine; ER: Entropy Regularization; EP: Entropy Pooling.

We compared the average training times of various models, as shown in Figure 4. The CNN with a regression task had the shortest training time at 35.6 min, while the CNN feature embedding methods with LSTM and SVM took slightly longer, at 37.8 and 36.1 min, respectively. Training times increased significantly with the use of Temporal Convolutional Networks (TCN), with the CNN+TCN model requiring 105.6 min. Introducing entropy pooling reduced this time to 85.7 min, and entropy regularization lowered it to 99.6 min. When both techniques were applied, the training time further dropped to 93.9 min.

## 5. Conclusions

In this paper, we introduced a novel framework for music groove rating that integrates Convolutional Neural Networks with Temporal Convolutional Networks, incorporating entropy regularization and entropy pooling techniques. The proposed architecture effectively addresses the challenges associated with accurately predicting groove levels by capturing long-range dependencies within musical time-series data and optimizing the model’s performance through advanced entropy-based methods.

The experimental results demonstrated that the integration of entropy pooling and regularization significantly enhances the predictive accuracy of the model. These findings indicate the effectiveness of our CNN–TCN architecture in capturing the complex patterns associated with musical groove.

However, it is important to acknowledge the limitations of our study. The current framework relies on a relatively narrow dataset, which may affect the generalizability of our results to a broader range of music styles. While our framework achieved notable improvements in groove prediction accuracy, future work could focus on expanding the dataset to include a wider variety of music genres.

## Figures and Tables

**Table 1:** Performance parameters of per-fold validation.

	MSE	R^2^
Fold 1	73.115	0.850
Fold 2	100.659	0.807
Fold 3	72.242	0.837
Fold 4	96.435	0.773
Fold 5	83.436	0.818
Mean ± SD	85.177 ± 11.681	0.817 ± 0.027

**Table 2:** Comparison of performance with other methods.

Dataset	Model	MSE	R^2^
	CNN+TCN+Entropy Pooling	89.879	0.804
	CNN+TCN+Entropy Regularization	99.837	0.783
	CNN+TCN+Entropy Pooling and Regularization	85.177	0.817
Janata	CNN+TCN	103.161	0.766
	CNN+Regression task	128.224	0.731
	CNN Feature Embedding+LSTM	107.200	0.707
	CNN Feature Embedding+SVM	253.094	0.548

## Data Availability

The code is available upon reasonable request to the corresponding.
